# Using epidemiological data to identify needs for child-rearing support among Chinese parents: a cross-sectional survey of parents of children aged 6 to 35 months in 15 Chinese cities

**DOI:** 10.1186/s12889-019-7635-y

**Published:** 2019-11-07

**Authors:** Yue Zhang, Matthew Sanders, Weiwei Feng, He Tang, Huishan Wang, Xi Jin, Jieling Wu, Guangwen Huang, Jin Sun, Yan Luo, Lanqiu Lv, Shuangqin Yan, Dongmei Zhao, Lijuan Mu, Dongmei Yan, Hong Wang, Xueting Gao, Jing Yang, Hong Wang, Nianrong Wang, Jie Shao, Jinliuxing Yang, Divna Haslam

**Affiliations:** 1 Children’s Health Care Department, National Center for Women and Children’s Health, Chinese Center for Disease Control and Prevention, Beijing, China; 2 Parenting and Family Support Centre, School of Psychology, the University of Queensland, Brisbane, QLD Australia; 3 Guangdong province Maternal and Child Health Hospital, Guangdong, China; 4 Hunan province Maternal and Child Health Hospital, Hunan, China; 5 Dalian Maternal and Child Health Hospital, Liaoning, China; 6 Guiyang Maternal and Child Health Hospital, Guizhou, China; 7 Ningbo Women and Children’s Hospital, Zhejiang, China; 8 Ma’anshan Maternal and Child Health Hospital, Anhui, China; 9 Qilu Children’s Hospital of Shandong University, Shandong, China; 10 Fangshan District Maternal and Child Health Hospital, Beijing, China; 11 Lianyungang Maternal and Child Health Hospital, Jiangsu, China; 12 Hubei Province Maternal and Child Health Hospital, Hubei, China; 13 Northwest Women and Children’s Hospital, Shanxi, China; 14 Qinghuangdao Maternal and Child Health Hospital, Hebei, China; 15 Sichuan province Maternal and Child Health Hospital, Sichuan, China; 16 Chongqing Maternal and Child Health Hospital, Chongqing, China; 17 The Children’s Hospital, Zhejiang University School of Medicine, Zhejiang, China

**Keywords:** Child rearing, Well-child care, Infants, Support seeking, Service delivery, Parenting, Child behavior

## Abstract

**Background:**

The quality of the family environment—in particular, the kind of parenting children receive in their early years—plays a critical role in influencing children’s growth and development. To facilitate the development and delivery of appropriate parenting and family interventions for Chinese parents, this study explores the prevalence of the difficulties that may arise in the course of child-rearing, the associated sociodemographic factors and parents’ help-seeking behavior.

**Methods:**

A cross-sectional self-reporting survey was conducted with a sample of 2229 parents of children between 6 and 35 months of age. Using a stratified random-digit design, parents from 15 Chinese cities were surveyed to determine their child-rearing difficulties, support-seeking behavior and their preferences for service delivery. The sociodemographic factors that influenced major child-rearing difficulties were analyzed using bivariate and logistic analyses.

**Results:**

The majority (87.5%) of Chinese parents of children aged 6–35 months reported experiencing child-rearing difficulties. Nearly one third (31.5%) of parents reported experiencing major difficulties. Feeding and sleep problems were most often reported. Regression analysis revealed that major child-rearing difficulties most often involved male children (OR = 1.35, 95% CI 1.11–1.64), single-child households (OR = 1.38, 95% CI 1.07–1.77), and households with financial problems (OR = 1.40, 95% CI 1.06–1.85). Just over one third of parents (33.44%) sought professional support, while 21.37% had attended a parenting course in the past year. Prefer ways of sourcing parental support included professional online platform (69.24%), self-help books (43.70%), face-to-face consultation (24.99%), and attending lectures (36.57%).

**Conclusions:**

Child-rearing difficulties are common among parents of children between 6 and 35 months of age in Chinese cities. The family with boys, single-child, financial problems, and father not joining in child-rearing may face the high risk to major child-rearing difficulties. The national initiative to provide more guidance and support for child-rearing difficulties is worthwhile, as is the development of online parenting programs.

## Background

The importance of nurturant care in the rearing of children has long been highlighted in the literature, particularly with regard to children up to 5 years of age [1]. The quality of such care is known to have a significant impact on many areas of a child’s life, including but not limited to areas such as language acquisition and communication, intellectual and emotional development, sibling and peer relationships, school performance, and overall health [2, 3]. Much of the research literature on child-rearing has focused on child-rearing practices [4], parents’ confidence [5], parenting stress [6] and parenting styles [7]. Unfortunately, the current literature yields few conclusions about parents’ child-rearing challenges and support-seeking behavior for parents of children under 3 years old, because most studies have focused on children aged 3–6 years old in western countries. Previous studies have identified evidence of variations in child-rearing in different cultural contexts, for example, the Chinese and immigrant Chinese parents tended to rate higher on parental control than Canadian toddlers’ parents, or Caucasian-American parents of children in kindergarten, Grade 1, and Grade 2 [8, 9]. This study sought to contribute to this literature by examining the difficulties and support-seeking regarding child-rearing by parents of children aged 6–35 months in Chinese urban areas.

Historically, a significant increase in parental concern regarding their children’s behavior and development has been noted during the 1960s, particularly regarding children below 5 years of age [10]. A similar phenomenon was reported by Chinese and Japanese studies [11, 12]. One 2009 report shown that 30.6% of Chinese households with children up to 3 years of age found child-rearing to be much more difficult than it used to be [11]. The proportion of Japanese mothers of 18-month-old babies who reported feeling stress during child-rearing had increased from 11% in 1980 to 33% in 2002 [12]. It is possible that this phenomenon may be partly due to the decreased morbidity and mortality from childhood infectious diseases and the rapid pace of social change as it affects family life in China and Japan, such as economic development, scientific progress, and improvements in communication at home and abroad. The changes in family structure, employment, and residence are all aspects of the chronosystem that may influence parenting (p537) [2]. This trend clearly highlights the importance of further exploration of current child-rearing in different cultural contexts.

Studies relating to the challenges and influencing factors of child-rearing are very limited. Some research show that feeding, sleeping, development, safety, and other parenting issues are common in families in Western countries, and different health concerns arise in different age groups [13, 14]. The mothers of children below 2 years of age were particularly concerned about the health, behavior, and communication [15]. And many such parents were also concerned about childhood diseases, health and nutrition, physical development, and child safety [14, 16]. Meanwhile, O’Brien and Marion (1996) found the most child-rearing difficulties reported by parents of infants and toddlers centered on children’s irritating behavior and tended to be reported by parents with more than one child [17]. About 53% of parents with children up to 3 years of age wanted information on at least three areas of parenting [15]. The topics that the parents discussed with pediatricians also varied with the child’s age, even in the age group below 3 years (e.g., encouraging learning is different for parents of a 3-month-old vs. a 2-year-old, and the concerns regarding sleeping patterns was decreased with age) [18, 19].

Individual characteristics and environment seem to play a significant role in child-rearing challenges, indicating that some families may need different or more intensive support. Garbutt (2012) found the parents had different concerns about child’s health among different child’s age-group, race/ethnicity, or parental income [14]. Other studies related to child-rearing problems focus on some special groups, such as, low birth weight (LBW) [20, 21], single motherhood [22], immigrant status [23], and race/ethnicity [13].. However, most of the factors in current literature are explored separately, without considering the influence by multiple factors. Culture also plays a critical role in child-rearing practices, as Graff (1995) emphasized [22]. And the results regarding western research cannot be simply generalized to the Chinese context. Specifically, cultural beliefs and practices may influence the simplest activities of child care, such as feeding, toilet training, discipline, and seeking health care. The views may concern the socialization agenda for children, the role of parents, and the accepted norms of behavior [24]. For example, Chinese caregivers were more worried about feeding, sleeping, and disease in child-rearing, while they were not so worried about cultivating children’s independence and peer relationships [11]. It was very common for grandparents to serve as primary caregivers for children of 0–2 years old in Chinese urban areas, accounting to 49.6–64.72% of cases [11, 16, 25]. Grandparents were more likely than parents to worry about their children not eating well, limit their gramdchildren’s activities, and believe some traditional opinions, such as “Giving children herbal medicines occasionally helps protect them from diseases” [11]. However, American children were more likely to be raised in two-parent households, more isolated from extended family members [26]. Concerning support-seeking, 56.3% of caregivers thought their current knowledge on and experience relating to parenting did not adequately address parents’ needs [11], while others Chinese studies have focused on certain specific aspects of child-rearing problems, such as feeding and sleeping [27, 28]. All of these Chinese studies were from single cities, and the problems of child-rearing remain unresolved.

The study of parents’ solutions to child-rearing difficulties and their support-seeking behavior would aid health care providers and policy makers to develop intervention programs. The most popular ways in which families hope to solve child-rearing problems in China were face-to face counseling (62.1%) and parenting lecture (57.8%) [11]. Thus, discussing with a professional and participating in parenting courses were the two professional forms of support-seeking we focused on, which are proved as effective approaches to provide services and closely related to the service of medical institutions. Since 2013, anticipatory guidance in child-rearing is recommended by a national guideline [29], which should be provided by primary care practitioners in community health service centers (CHSC) in China. According to 2012 data, approximately 87% of children below 3 years of age were regularly seen by primary care practitioners to monitor their growth and development [30]. However, some studies reported that most pediatricians and other health care providers in China do not have enough time to deliver appropriate well-child checkups [15]. Meanwhile, the intervention of child-rearing problems could be targeted effectively through an online program [31], but no evidence supporting this claim has been found in Chinese parents.

This study is the first of a series presenting the findings from a larger survey of urban Chinese parents of children less than 6 years of age. The research team was consisted of 5 researchers from child health, epidemiology and health statistics, and 16 investigators were trained. In total, 4721 parents completed the survey between August and October 2017. This study focused specifically on the analysis of the common child-rearing problems and solutions experienced by the parents of children aged 6 to 35 months. The main aims were as follows: (1) to establish the prevalence of specific parenting problems and challenges among parents living in the urban areas of China, (2) to examine the impact of a range of demographic factors as predictors of major parenting challenges with a view to identifying those parents in most need of help, and (3) to investigate the parents perceived to be in need of professional parenting support and their preferences for service delivery, thus helping us to learn exactly what child health care providers ought do to support these parents. Therefore, we hypothesized that child-rearing difficulties are prevalent among parents in China, and Chinese parents of children up to 3 years of age have their own special child-rearing problems, which are influenced by some sociodemographic factors. We also postulate that parents with major parenting difficulties should have a higher need for professional counseling and parenting courses, and that provision of online sources for parenting support will be an effective way for parents of infants and toddlers to solve child-rearing problems. The survey was conducted into the type and severity of child-rearing difficulties and parents’ help-seeking behavior with a view to facilitating the development and evaluation of child health care services.

## Methods

### Design and sampling

A cross-sectional survey methodology was used. The survey recruited participants in 15 cities located in 14 out of a total of 31 provinces in China, which included eight eastern cities, three central cities, and four western cities (according to the Health Statistics Yearbook of China [32]).Firstly, the maternal and children’s hospitals of every province were contacted by the research team to join our study. Maternal and children’s hospitals in 15 cities agreed to enroll in our study. The hospitals who agreed to partner would be in coordinating the administration of the surveys, because they closely contact with the local CHSC and provide technical guidance for CHSC. The recruited hospitals provided the overall directory of district and community and numbered them. Using a stratified random-digit design for sampling, one district was randomly selected from each city and one community was randomly selected from each district. In China, the CHSC supplies free health checkups and immunization regularly for children under 6 years old, and the general information of records serve as the basis for children enrollment in this study. The free health checkups for children include two home-visiting for newborns (at 7 and 28 days), eight health checkups for infant and toddler (at 3, 6, 8, 12, 18, 24, 30, and 36 months),and yearly checkups for children 3–6 years of age. According to the children’s list in CHSC, all children less than 3 years of age in the community were stratified by age group (6 months to < 1 years, 1 year to < 2 year, and 2 years to < 3 years). The sample size of this study was 2080, according to the calculation based on the estimated prevalence rate of childrearing problems (1/3), permissible error (0.15), one-side α (0.05), and stratified by gender and age groups. Considered data missing, 25 boys and 25 girls were randomly chosen as sample in each age group, for a total of 150 children in each community. The public health doctors informed parents of the survey by phone or face-to-face. The parents were asked some questions to determine if they met the inclusion criteria and whether they would like to participate. If someone refused or did not meet the requirements, the sample had to be randomly extracted again. Inclusion criteria: (1) the parents had a child or children between 6 months and 3 years of age without any major illness, (2) the family had a local household registration or had resided in the city for more than a year. Exclusion criteria: (1) the children had severe developmental problems or congenital diseases or (2) the parents were unable to read and write at an acceptable level. The data were collected from questionnaires completed by the parents in writing. These were administered when the parents took their child to the CHSC for health checkups after they agreed to join in this study. The final response rate was 94.05%. The high response rate may be related to the fact that the doctor informed parents in advance or established a good relationship during the long-term health checkups. The most common reasons for participant rejection were that they were absent during this period, the child was ill, or their availability was restricted by time constraints. Nine questionnaires were labeled as incomplete. The total sample size of this survey was 2229 children aged 6 to 35 months. Details of the sample are given in the results section.

### Measurements

Variables assessed in this study were obtained from self-report questionnaires, including social demographic details, reports of child-rearing problems, and history of support-seeking. The parent-report questionnaire was designed specifically for the parents of children less than 3 years of age. It took approximately 5–10 min to complete.

Social demographic variables included both child and family variables (see Table 1). Child variables included the child’s age, gender, perinatal risks (e.g., delivery mode, prematurity, low birth weight, and so on). Family variables included the mother’s age, mother’s education level, family structure, economic burden, and so on. The cutoff value of each family’s annual income (15,000 USD) close to the average annual income in urban families, which was obtained by multiplying the average annual income pre capita in urban areas by the average number of people per household according to data from 2017 [33]. The status of primary caregivers was also reported in this study using a multiple-choice question, which was consistent with the situation that Chinese families may have multiple primary caregivers. Primary caregivers could include parents, families and other people who are directly responsible for the child at home, and also include carers outside the home, such as people working in organized day care [34].

**Table 1:** Demographic characteristics of entire parents sample

Characteristic	Subgroup	Number(N)	Percentage(%)
Child’s Age-groups (*N* = 2229)	6 m~ <1y	730	32.75
	1 y~ < 2y	762	34.19
	2 y~ < 3y	737	33.06
Child’s Gender (N = 2229)	Boy	1129	50.65
	Girl	1100	49.35
Mother’s Age (N = 2225)	≤ 25 year	197	8.84
	26–30 year	1056	47.38
	31–35 year	663	29.74
	36–40 year	265	11.89
	≥41 year	44	1.97
Mother’s Education Level (*N* = 2227)	Completed junior high school or less	163	7.31
	Completed senior high school or technical certificate	422	18.93
	University and college degree	1449	65.01
	Graduate degree	193	8.66
Perinatal Factors			
Caesarean section (*N* = 2219)	Yes	932	42.00
Premature (*N* = 2208)	Yes	137	6.20
Low birth weight (*N* = 2204)	Yes	87	3.95
Children number in family (*N* = 2217)	One-child	1518	68.47
	Multiple children	699	31.53
Family structure^a^ (*N* = 2224)	Nuclear family	1041	46.80
	Extended family	1158	52.07
	Single parent family	25	1.12
Primary Caregivers			
Mother (N = 2225)	Yes	1670	75.06
Father (N = 2225)	Yes	693	31.15
Grandparent (*N* = 2225)	Yes	1276	57.35
Other (N = 2225)	Yes	124	5.57
Family’s annual income (USD, *N* = 2205)	<15,000	832	37.33
	≥15,000	1372	61.60
Unable to meet expenses (*N* = 2074)	Yes	302	14.56

^a^ Nuclear family: parents and their minor children live together. Extended family: include stem family and joint family. Extended family means grandparents and/or parents’ brothers or sisters, live together with parents and children. Single parent family: one parent lives together with their minor children, including the family which parents lived in two areas

*Measures of child-rearing problems:* Because there is no standardized measurement of child-rearing problems, the questionnaires were developed specifically for the purpose of the present study. The recruited parents were asked whether they had encountered child-rearing problems over the preceding 3 months and how difficult it was for them to deal with it. Three to five specific problems in each category were nominated by the research team. A total of 22 items were merged into 19 items according to the suggestion of clinical and research experts of developmental pediatrics, child psychology, and child health (Table 2). Cronbach’s α was 0.871, and fluctuated in 0.637–0.795 for these categories. Child-rearing problems were ascertained via several yes/no questions in the survey. For example, “Did you experience problems feeding your child?” If one chose yes, she or he would then have to answer the question “Did you have difficulty in dealing with the feeding problems?” The respondents used a 3-point categorical scale to indicate how difficult the behavior was to deal with. They were “no difficulty at all (0)”, “some difficulty (1)”, and “a great deal of difficulty (2)”.To facilitate the analysis of these replies, they were then combined into a summary variable of having any child-rearing difficulty. If a respondent answered yes to at least one of the items, the situation would be categorized as having child-rearing difficulties. Parents who reported that they had a problem about child-rearing (yes) and no difficulty (0) in dealing with it were identified as having minimal child-rearing difficulties. Parents who reported that they had a problem about child-rearing (yes) and some difficulty (1) in dealing with it were identified as having moderate child-rearing difficulties. Parents who reported that they had problems about child-rearing (yes) and great difficulty (2) in dealing with it were identified as having major child-rearing difficulties. If a respondent named any one item as a major difficulty, they were placed in the major child-rearing difficulties group.

**Table 2:** Proportion of specific aspects in parents with child-rearing problems

Problems	Specific aspects	N	Major difficulties (%)	Moderate difficulties (%)	Minimal difficulties (%)	No Difficulties (%)
Feeding	Unwilling to eat by herself/ himself	2212	5.29	21.62	15.87	58.36
	Eat less	2212	4.38	23.98	18.37	53.51
	Picky eater	2212	4.14	24.31	18.16	53.71
	Not drink milk	2212	3.27	8.67	24.14	63.84
Sleeping	Sleep with milk or eating before bed	2202	5.77	17.39	20.89	55.77
	Patting or holding to sleep	2202	4.22	15.84	19.66	59.45
	Can’t get to sleep by herself/ himself when wake up at night	2202	3.91	11.25	19.12	65.67
	Sleep in day-time and awake at night	2202	2.00	8.90	20.80	68.26
Development	Language	2178	4.32	21.69	16.57	58.36
	Gross motor	2180	2.20	9.81	22.02	66.19
Behavior	Difficulties in car	2184	2.24	18.27	15.98	61.81
	Disobey	2190	2.15	28.79	14.52	53.15
	Always interfere with others	2177	1.19	16.51	15.62	65.64
	Hurting Others	2185	0.55	4.93	17.44	76.89
Emotional	Separation anxiety	2180	4.13	20.43	15.23	59.27
	Tantrums	2189	2.56	18.62	15.53	62.36
	Keep crying	2171	1.47	16.55	15.89	65.59
Take care	Take care of more than one child at a time	2163	1.11	10.89	15.30	71.80
Others		2229	0.30	3.80	12.30	83.70

*Questions of help-seeking:* To assess what support they needed to deal with their child-rearing problems, this part included four questions. The question stems were consistent with the International Parenting Survey (IPS, Morawska, Heinrichs, & Sanders, 2011), which was developed as a tool to inform the tailoring of parenting programs to meet parental needs and preferences, including questions about parents’ perceptions of their need for help in dealing with the child’s behavior, their current use of services to address identified issues, their awareness of and participation in parenting programs, and similar issues [35, 36]. However, some options were modified based on the purpose of the present study. Two questions in this survey, whether they had discussed them with a professional and whether they had participated in a parenting course during the previous 12 months, were modified into yes/no from multiple-choice, because the name of specific parenting program as options in IPS are not familiar to Chinese parents. In addition, the nonparticipating parents were asked to explain why they had not participated in such a course by answering a multiple-choice question from IPS (Table 5). One option named“culturally inappropriate strategies used”was removed, because the pre-survey indicated that the participants found it difficult to understand, even this item was expressed in different ways and words. For preferred ways of parenting support, parents were asked to name their preferred delivery formats from a range of options by a multiple-choice question, including self-help books, professional online platform, calling consultation, attending lectures, group classes, face-to-face consultation, workplace access, home visits and others.

### Statistical analysis

Sociodemographic variables, the incidence of child-rearing problems and help seeking were described by frequencies and percentages. Bivariate associations between the sociodemographic variables and the major child-rearing problems were evaluated using the chi-square test. Logistic regression was used to examine the association between the sociodemographic background and major child-rearing challenge measures in multivariate analysis: the dependent variable was major child-rearing difficulties (yes/no), and independent variables included the child’s age, gender, delivery mode, prematurity and LBW, the mother’s age and education level, the family structure, the family’s annual income, and primary caregiver (Table 1). The child’s age group, mother’s age group and mother’s education level, which were ordered multi-categorical variables, were translated into dummy variables in logistic analysis. The differences with regard to participating in parenting courses and reasons for not participating between parents with major difficulties and other parents were also evaluated using the chi-square test. Odds ratios (ORs) were presented as results for both bivariate associations and logistic regression model. All of the data preparation and statistical analyses were performed using the SPSS for Windows software program (version 18.0).

## Results

### Demographics

In total, 2229 parents of children aged 6 to 35 months completed the questionnaires. The study population was mainly mothers, account to 81.38%. About half of the children were male and the percentages of each age group were almost equal. Nearly two thirds (65.01%) of mothers had completed tertiary education, and nearly half (47.38%) were aged between 26 and 30 years. The demographic characteristics of the participants in this study are presented in Table 1.

### Prevalence of child-rearing problems

In this study, the vast majority of parents (87.53%, or 1826/2086) reported that they had experienced at least one child-rearing problem during previous 3 months, and close to one-third (31.59%, or 659/2086) reported major child-rearing difficulties. The most common child-rearing problems, encountered by 50 to 60% of parents, were feeding problems (63.43%, or 1403/2212), sleeping problems (60.72%, or 1337/2202), behavioral problems (56.78%, or 1252/2205), emotional problems (55.14%, or 1202/2180), and developmental problems (49.21%, or 1090/2215).

The severity of child-rearing problems in Chinese parents is shown in Table 2. The incidences of specific child-rearing problems varied from 16.30 to 46.85%. The top major child-rearing difficulties were as follows: child unwilling to sleep without milk/food before bed (5.77%), child unwilling to eat by herself or himself (5.29%), child eating less (4.38%), language problems (4.32%), needing to be patted or held in order to fall asleep (4.22%), picky eater (4.14%), separation anxiety (4.13%), and child cannot get back to sleep alone after waking up at night (3.91%).

### Factors related to major child-rearing difficulties

The relations between major child-rearing problems and the sociodemographic factors were examined by bivariate analyses and logistic regression analysis. The bivariate analyses results with regard to the factors related to major child-rearing difficulties are shown in Table 3. Of parents of boys, 33.78% were likely to report major child-rearing difficulties as compared with 29.38% of parents of girls. The father as main caregiver was less likely to report major child-rearing difficulties (27.93% yes vs. 33.26% no). The families with a single child (33.78% yes vs. 26.67% no) were significantly more likely to report major child-rearing difficulties, so did in families being unable to meet expenses (37.82% yes vs. 31.20% no).

**Table 3:** Variables Associated with Major Child-rearing Difficulties

Variable		Major Parenting difficulty		Bivariate analyses	
		Yes	No	*P*	OR(95% CI)
Child’s Age-group	6 m ~ <1y	207 (30.40)	474 (69.60)	0.41	0.92 (0.76–1.2)
	1y~ <2y	245 (34.03)	475 (65.97)	0.08	1.19 (0.98–1.44)
	2y~ <3y	207 (30.22)	478 (69.78)	0.35	0.91 (0.75–1.11)
Child’s Gender	boy	354 (33.78)	694 (66.22)	0.03	1.23 (1.02–1.48)
	girl	305 (29.38)	305 (70.72)		
Mother’s Age	≤25y	53 (29.61)	126 (70.39)	0.55	0.90 (0.65–1.26)
	26-30y	328 (33.06)	664 (66.94)	0.17	1.14 (0.95–1.37)
	31-35y	199 (31.94)	424 (68.06)	0.82	1.02 (0.84–1.25)
	36-40y	70 (28.11)	179 (71.89)	0.21	0.83 (0.62–1.11)
	≥41y	9 (21.43)	33 (78.57)	0.15	0.56 (0.28–1.23)
Mother’s Education Level	completed junior high school or less	41 (26.62)	113 (73.38)	0.17	0.77 (0.53–1.12)
	completed senior high school or technical certificate	116 (30.53)	264 (69.47)	0.62	0.94 (0.74–1.20)
	university and college degree	444 (32.48)	923 (67.52)	0.23	1.13 (0.93–1.37)
	graduate degree	57 (31.15)	126 (68.85)	0.89	0.98 (0.71–1.36)
Perinatal Factors					
Delivery mode	natural birth	371 (31.05)	824 (68.95)	0.53	0.94 (0.78–1.14)
	cesarean section	286 (32.35)	598 (67.65)		
Premature	yes	40 (32.52)	83 (67.48)	0.79	1.05 (0.71–1.56)
	no	610 (31.38)	1334 (68.62)		
Low birth weight	yes	32 (39.02)	50 (60.98)	0.13	1.42 (0.90–2.24)
	no	615 (31.01)	1368 (68.99)		
Primary Caregivers					
Mother	yes	491 (31.37)	1074 (68.63)	0.69	1.04 (0.84–1.29)
	no	167 (32.30)	350 (67.70)		
Father *	yes	181 (27.93)	467 (72.07)	0.02	0.76 (0.63–0.95)
	no	477 (33.26)	957 (66.74)		
Grandparent	yes	384 (32.32)	804 (67.68)	0.42	0.93 (0.77–1.12)
	no	274 (30.65)	620 (69.35)		
Families status					
Single-child family*	yes	483 (33.78)	947 (66.22)	0.00	1.40 (1.14–1.72)
	no	172 (26.67)	473 (73.33)		
Nuclear family	yes	312 (31.64)	674 (68.36)	0.94	1.01 (0.84–1.21)
	no	345 (31.48)	751 (68.52)		
Single-parent family	yes	9 (37.50)	15 (62.50)	0.53	1.31 (0.57–3.00)
	no	648 (31.49)	1410 (68.51)		
Extended family	yes	336 (31.34)	736 (68.66)	0.83	0.98 (0.84–1.18)
	no	321 (31.78)	689 (68.22)		
Family’s annual income	< 15,000 USD	246 (31.70)	530 (68.30)	0.97	1.00 (0.83–1.22)
	≥15,000 USD	409 (31.61)	885 (68.39)		
Unable to meet expenses *	yes	107 (37.81)	176 (62.19)	0.03	1.34 (1.03–1.74)
	no	522 (31.20)	1151 (68.80)		

* Significance *p* < 0.05

The regression analysis results are shown in Table 4, the factors that remained in the final model that were positively associated with major child-rearing difficulties were boys (OR = 1.35, 95% CI 1.11–1.64), single-child households (OR = 1.38, 95% CI 1.07–1.77), and unable to meet expenses (OR = 1.40, 95% CI 1.06–1.85). Factors that were negatively associated with major child-rearing difficulties were father as main caregiver (OR = 0.77, 95% CI 0.61–0.98). There was no significant difference between major difficulties and child’s age group, mother’s age, mother’s education level, perinatal factors, family’s annual income, family structure.

**Table 4:** Model for logistic regression of parents with major child-rearing problems as predicted by child’s, maternal and family status (*N* = 1895)

	B	SE	Wald	*P*.	OR	95% CI	
Child’s age-group (base:6 m ~ <1y)							
1y~ <2y	0.02	0.13	0.03	0.87	1.02	0.80	−1.31
2y~ <3y	−0.21	0.12	2.89	0.90	0.81	0.64	−1.03
Child’s Gender (base: girl)	0.30	0.10	8.86	0.00	1.35	1.11	−1.64
Mother’s Age (base: ≤25y)							
26-30y	−0.14	0.44	0.09	0.76	0.88	0.37	−2.01
31-35y	−0.30	0.40	0.56	0.46	0.74	0.34	−1.63
36-40y	−0.33	0.40	0.69	0.41	0.72	0.33	− 1.57
≥ 41y	−0.29	0.41	0.51	0.48	0.75	0.33	−1.68
Mother’s Education Level (base: Completed junior high school or less)							
Completed senior high school or technical certificate	0.31	0.29	1.15	0.28	1.36	0.76	−2.39
University and college degree	0.03	0.22	0.01	0.91	1.03	0.67	−1.58
Graduate degree	−0.03	0.18	0.03	0.86	0.97	0.68	−1.38
Perinatal Factors (base: no)							
Cesarean section	−0.04	0.10	0.16	0.69	0.96	0.78	−1.17
Premature	−0.05	0.24	0.05	0.83	0.95	0.60	−1.50
Low birth weight	0.42	0.27	2.33	0.13	1.52	0.89	−2.60
Primary Caregivers (base: no)							
Mother	0.14	0.14	1.08	0.30	1.15	0.88	−1.51
Father	−0.26	0.12	4.63	0.03	0.77	0.61	−0.98
Grandparent	0.00	0.13	0.00	0.99	1.00	0.78	−1.28
Family structure (base: Nuclear family)							
Extended family	0.22	0.46	0.22	0.64	1.24	0.50	−3.08
Single parent family	0.22	0.46	0.22	0.64	1.24	0.50	−3.06
Single-child family (base: no)	0.32	0.13	6.33	0.01	1.38	1.07	−1.77
Family’s annual income (base: no)	−0.02	0.11	0.04	0.85	0.98	0.79	−1.22
Unable to meet expenses (base: no)	0.34	0.14	5.54	0.02	1.40	1.06	−1.85

# Model goodness of fit:-2 Likelihood =2329.13, R squared = 0.028. X^2^ = 38.49, *p* = 0.02

### Seeking of parenting support

To explore the help-seeking of parenting support, the parents were specifically asked to indicate whether they had discussed them with a professional and whether they had participated in a parenting course in the past year. One-third (33.44%, or 740/2213) of parents reported that they had discussed their child-rearing problems with a professional person (including pediatricians, nurses, social workers, parenting practitioners, public health providers, and others). More than one-fifth (21.37%, or 473/2213) of parents attended a class on child development, child behavior, or parenting. According to the chi-square analysis results, there were no significance differences with regard to participating in parenting courses between parents with major difficulties and other parents (30.30 and 32.02%, OR = 0.98, 95% CI: 0.73–1.15).

Reasons given for not participating in parenting courses are presented in Table 5. The most common reasons were lack of time (50.62%) and inconvenient timing of services (36.40%). The parents with major child-rearing difficulties were more likely to choose financial cost (OR = 1.45, 95% CI: 1.03–2.04), conflict with job (OR = 1.34, 95% CI: 1.03–1.74) and lack of awareness of any programs (OR = 1.55, 95% CI: 1.06–2.26) as the reason for not participating in parenting courses. There were no other significance differences for other reasons between the two groups of parents (those with major childrearing problems and those without).

**Table 5:** The bivariate analysis of the relationship between reasons for non-participation-training and major child-rearing difficulties (*N* = 1610, %)

	Total	Major Child-rearing Difficulties			OR	95% CI
		Yes	No			
Lack of time	815 (50.62)	273 (52.91)	542 (49.54)		1.14	0.93–1.41
Inconvenient timing of services	586 (36.40)	193 (37.40)	393 (35.92)		1.07	0.86–1.32
Conflict with my job *	294 (18.26)	110 (21.32)	184 (16.82)		1.34	1.03–1.74
Didn’t feel that needed to	262 (16.27)	77 (14.92)	185 (16.91)		0.86	0.65–1.15
Financial cost *	156 (9.69)	62 (12.02)	94 (8.59)		1.45	1.03–2.04
Wasn’t aware of any programs *	121 (7.52)	50 (9.69)	71 (6.49)		1.55	1.06–2.26
Transport difficulties	121 (7.52)	47 (9.11)	74 (6.76)		1.38	0.94–2.02
used to learned but it no use	68 (4.22)	20 (3.88)	48 (4.39)		0.88	0.52–1.50
Extended family or partner not supportive	20 (1.24)	9 (1.74)	11 (1.01)		1.75	0.72–4.24
Felt uncomfortable accessing a parenting program	6 (0.37)	2 (0.39)	4 (0.37)		1.06	0.19–5.81
Other	103 (6.40)	29 (5.62)	74 (6.76)		0.82	0.53–1.28

* *P* < 0.05

Parents’ views with regard to accessing services were determined by asking about their personal preferences with a multiple-choice question. About 69.24% of parents reported that a professional online platform would be the best way accessing help, followed by reading a self-help handbook on parenting (43.70%), seeking a face-to-face consultation with a specialist (24.99%), and attending lectures (24.16%). However, phone consultation (17.09%), home visits (14.78%), workplace access (7.90%), and group classes (7.44%) were still rejected by most parents. No statistically significant differences in delivery modes were found between parents with major child-rearing difficulties and other parents.

## Discussion

This study showed the prevalence, the degree, and the related factors of child-rearing problems for parents of children 6–35 months of age and the support-seeking behavior of parents in urban areas of China. It is the first to provide relatively comprehensive evidence of child-rearing difficulties among Chinese parents with a large sample size, and will be useful for developing an approach to screen parents with major child-rearing difficulties that is applicable to the entire population of China and where families with higher needs are able to access higher levels of support to promote child health and development in both China and other Asian countries.

One of the goals of this study was to establish the prevalence of specific child-rearing problems among parents of young children living in the urban areas of China. This study found a high rate of parents (87.53%) struggling with issues related to child-rearing for children aged 6–35 months, but not surprising. The result is similar to that of another study, which finds 11.8% of Chinese caregivers of children aged below 3 years reporting no parenting difficulties [11]. In fact, our study extends this finding by demonstrating a series of specific problems and the levels of difficulty of childrearing in China urban areas. The most common child-rearing problems of parents were basic needs, such as feeding and sleeping problems, which nearly two thirds in children up to 3 years old respectively. Parenting tasks in the first 3 years can be viewed on a continuum in which the early months are focused on physical care and safety of infant, with socialization following behind as an important secondary focus [2]. Almost two-thirds of Americans believed that most parents face the same child-rearing challenges several times before they really seek help raising their children [27]. These findings indicate that the challenges of child-rearing are common and probably normal, because every child is unique and most new parents lack parenting experience even when they come from in multi-children families.

The factors influencing childrearing problems in this study have been systematically confirmed, and they are partly consistent with the hypothesis. First, parents of boys and single-child households were more likely to encounter difficulties than were other parents in China, which may be related to the boys’ characters, more stress on new parents, the traditional ideas of great importance of boys, and aftereffects of China’s now-cancelled one-child policies. Second, the results of this study also highlight the father’s role in child-rearing. Although fathers are considered as the person who earns a living for the family [37, 38], the involvement of the father in parenting can reduce the stress and workload for mother [39], affect the quality of the parent-child relationship, decrease conflict, improve the quality of parenting, and modify child behavior. Third, contrary to the expectations of some, the major child-rearing problems were not significantly influence by the mother’s education level. Similar with Glascoe’s reviewed (2010), namely, that parents are equally able to raise important concerns regardless of differences in education and child-rearing experience [40]. Finally, the factors of childrearing problems in this study were extended to perinatal risks, such as delivery mode, prematurity and LBW. LBW and single-parent families appeared to have no effects on child-rearing problems in this study, which may be related to the relatively small sample size. Although the study found some factors influencing major childrearing problems significantly, the regression analysis with the low model goodness of fit, suggest that further research is required to identify other important factors influencing major childrearing problems in future.

One goal of this study, that is, assessing the help-seeking behavior among parents with child-rearing problems, has partially been achieved. As expected, the most prefer avenue for seeking support was online approaches, which can to mitigate many barriers commonly associated with lack of participation, such as lack of time, inconvenient timing of services and conflict with a job, as reported in this and other studies [30, 41]. The parents with major child-rearing difficulties in this study were more likely to choose financial cost, conflict with job and lack of awareness of any programs as the reason for not participating in parenting courses. Therefore, the development of professional, cost-friendly, and web-based approaches may be the most realistic and effective option to support parents with childrearing problems.

This study failed to detect significantly differences in consulting professional or participation in parenting courses by parents with major difficulties or other parents. It may be partially due to the parental perception, the practice and quality of counseling or parenting courses, and some other reasons need to be explored. Anticipatory guidance is a common and effective way for health care providers to help parents deal with issues such as feeding and nutrition, language development, discipline, and sleeping [18, 42]. However, a perceived lack of time and training are frequently cited barriers to the anticipatory guidance for potential problems [41]. The parents of children under 3 years old have at least 2 opportunities each year to consult health care providers at the time of health checkups, but only a third parents reported that they had discussed their child-rearing problems with a professional person in past year. So it is necessary to estimate the implementation of anticipatory guidance, and provide more guidance and support for child-rearing difficulties by national initiatives. In fact, individualized anticipatory guidance is not necessarily more time-consuming, and likely to be more effective in addressing parent needs’ when it is tailored to their particular needs and interests [14, 43]. With regard to parenting courses, this study found that nearly one fifths of parents reported attending the parenting courses, which is lower than that in an American’s survey (one-third, p110) [28]. A few pilot studies on parenting programs have been carried out or evaluated in China [42, 44], but no evidence-based parenting support program has yet been promoted on a large-scale nationwide. Most of parenting courses reported by parents may be loose, casual, and lacked any evidentiary support. Therefore, it is necessary to develop high-quality, evidence-based parenting programs that could meet the needs of parents in China.

Although this survey provides a snapshot of current child-rearing problems among urban families, some potential limitations should be noted in interpreting the results. First, the cities in this study were recruited, which may have introduced some selection biases; that is, the conclusions may not be generalized to other populations. Despite the potential bias, the data are comparable with national statistics, such as child’s gender [45], rates of cesarean section delivery [46], and proportion of two-parent families. Additionally, the use of self-report measures, though common in the field, is susceptible to potential bias in terms of selective reporting or recall bias. Moreover, the sample in this study excluded caregivers who could not read. It may be important to study such poorly educated caregivers, as it could have an influence on the mother’s knowledge and attitudes toward her child. It is essential that future research efforts include a larger number of samples so as to establish the reliability and validity of the questionnaires and the results.

## Conclusions

Child-rearing difficulties are common among parents who have children between 6 and 35 months of age in Chinese cities. The family with boys, single-child, having financial problems, and father not joining in child-rearing may face the high risk to major child-rearing difficulties. The children’s healthcare service providers and health policy makers should address the needs of these parents, particularly those from disadvantaged families. The national initiatives to provide more guidance and support for commonly-recognized child-rearing difficulties is worthwhile, as is the development and testing of online parenting programs.

## Data Availability

The datasets used and/or analyzed during the current study are available from the corresponding author on reasonable request.

## Abbreviations

CHSC: Community Health Service Centre
IPS: International Parenting Survey
LBW: Low Birth Weight
